# Complete Genome Sequence of a Putative Densovirus of the Asian Citrus Psyllid, *Diaphorina citri*

**DOI:** 10.1128/genomeA.00589-16

**Published:** 2016-07-28

**Authors:** Jared C. Nigg, Shahideh Nouri, Bryce W. Falk

**Affiliations:** Department of Plant Pathology, University of California Davis, Davis, California, USA

## Abstract

Here, we report the complete genome sequence of a putative densovirus of the Asian citrus psyllid, *Diaphorina citri*. *Diaphorina citri* densovirus (DcDNV) was originally identified through metagenomics, and here, we obtained the complete nucleotide sequence using PCR-based approaches. Phylogenetic analysis places DcDNV between viruses of the *Ambidensovirus* and *Iteradensovirus* genera*.*

## GENOME ANNOUNCEMENT

A metagenomic survey of viruses associated with *Diaphorina citri* revealed contigs displaying similarity to densovirus (DNV) structural (VP) and nonstructural (NS) genomic regions (1). These contigs were reported as genomic fragments of the tentatively named *Diaphorina citri* densovirus (DcDNV). To expand on the genomic sequence of DcDNV, DNA from *D. citri* collected in Taiwan was used for PCR, with primers designed to amplify the unknown sequence between the NS and VP coding regions, followed by Sanger sequencing. Sequence analysis revealed that DcDNV has an ambisense genome organization, with 25 nucleotides (nt) separating the VP and NS cassettes. Additional PCR-based strategies and Sanger sequencing were used to obtain the sequences at the extremities of the genome, which contain 210-nt inverted terminal repeats (ITRs) predicted to form simple 210-nt hairpins characteristic of subgroup B ambisense DNVs (2–4).

The complete genome sequence of DcDNV is 5,071 nt and contains four predicted open reading frames (ORFs). ORF1 (nt 300 to 1598) has a coding capacity of 432 amino acids (aa). BLASTp analysis of the full-length putative protein encoded by ORF1 indicates similarity with uncharacterized insect proteins (accession numbers XP_011214328.1 and XP_003248352.1). ORF2 (nt 338 to 2377) begins with a TTG codon at position 338 and is present in a -1 reading frame relative to ORF1. BLASTp analysis of the putative 679-aa protein encoded by ORF2 indicated the highest identity with *Cherax quadricarinatus* densovirus NS1 (query coverage, 67%; identity, 34%) (accession no. YP_009134732.1). Additionally, the ORF2-encoded protein possesses the rolling-circle replication initiator and helicase superfamily 3 motifs characteristic of DNV NS1 proteins (5–7). We did not identify DNV NS2 or NS3 ORFs. Among ambisense DNVs, lack of an NS3 ORF has been reported only for *Myzus persicae* densovirus (8). Although the phylogenetic position of DcDNV is unclear, phylogenetic analysis based on the NS1 amino acid sequence places DcDNV in an intermediate position between the subgroup B ambisense DNVs and the iteradensoviruses. Indeed, the organization of the NS cassette of DcDNV resembles that of iteradensoviruses more than other ambisense DNVs.

The VP cassettes of ambisense DNVs are on the complementary strand of that containing the NS cassettes and encode four or five structural proteins from one or two ORFs, respectively (4). ORF3 (nt 2402 to 4168) encodes a putative 588-aa protein that displays the highest identity to the VP1 protein of densovirus SC1065 based on BLASTp analysis (query coverage, 37%; identity, 31%). ORF4 (nt 4149 to 4766) has a coding capacity of 205 aa and encodes a putative protein containing the HDXXY and YXGXG phospholipase A2 motifs characteristic of DNV VP1 proteins (9). The full-length putative protein encoded by ORF4 shows the highest similarity with *Periplaneta fuliginosa* densovirus VP1 based on BLASTp analysis (query coverage, 43%; identity, 42%) (accession no. BAA82965.1). The structural proteins of ambisense DNVs are generated by leaky scanning and/or alternative splicing mechanisms. Splice site prediction using NNSPLICE (version 0.9) (10) indicates seven potential splicing donor sites and four potential splicing acceptor sites within the VP cassette.

### Nucleotide sequence accession number.

The GenBank accession number of the complete nucleotide sequence of DcDNV is KX165268.
